# Paradoxical Coronary Embolism Through a Patent Foramen Ovale Presenting as ST Elevation Myocardial Infarction With Distal Right Coronary Artery Occlusion

**DOI:** 10.7759/cureus.107188

**Published:** 2026-04-16

**Authors:** Cristina Suarez Chiriboga, Tambi Isaac, Roxana Lazarescu, Samy Matta

**Affiliations:** 1 Internal Medicine, Wyckoff Heights Medical Center, New York, USA; 2 Internal Medicine, Alexandria University, Alexandria, EGY

**Keywords:** coronary artery embolism, intravascular ultrasound, non-atherosclerotic myocardial infarction, paradoxical embolism, patent foramen ovale, st elevation myocardial infarction

## Abstract

We report the case of a 57-year-old man presenting with inferior ST elevation myocardial infarction. Initial electrocardiography demonstrated ST segment elevation in the inferior leads. After administration of aspirin and intravenous heparin, repeat electrocardiography showed partial improvement in ST segment elevation. Emergent coronary angiography revealed abrupt distal occlusion of branches of a dominant right coronary artery (RCA) with preserved proximal flow. Intravascular ultrasound (IVUS) demonstrated minimal coronary atherosclerosis without plaque rupture or dissection.

Transthoracic and transesophageal echocardiography (TEE) demonstrated a patent foramen ovale with atrial septal aneurysm and right-to-left shunting. Hypercoagulable evaluation was unremarkable. Follow-up echocardiography identified a small left ventricular apical thrombus.

## Introduction

Coronary artery embolism is an uncommon but clinically significant cause of acute myocardial infarction (AMI) and remains frequently underrecognized in routine clinical practice. In a large observational cohort, Shibata et al. reported that coronary artery embolism accounted for approximately 2.9% of acute myocardial infarction cases and demonstrated distinct angiographic characteristics compared with plaque-mediated infarction, including distal coronary occlusion and minimal underlying atherosclerosis [1]. Similarly, Raphael et al. highlighted that embolic myocardial infarction often presents with preserved proximal coronary flow and limited plaque burden, features that may help distinguish embolic occlusion from traditional atherosclerotic plaque rupture during coronary angiography [2].

Paradoxical embolism through a patent foramen ovale represents a recognized mechanism of systemic arterial embolization. Windecker et al. described how venous thrombi may bypass pulmonary filtration through right-to-left intracardiac shunting and enter the systemic circulation, producing embolic events in the brain, coronary arteries, or peripheral vasculature [3]. The presence of an atrial septal aneurysm may further increase embolic risk. Mas et al. demonstrated that increased septal mobility associated with atrial septal aneurysm facilitates thrombus passage across the interatrial septum and is associated with a higher incidence of paradoxical embolic events in patients with patent foramen ovale [4].

In addition to structural cardiac abnormalities, occult atrial fibrillation represents an important potential source of embolic events. In the Cryptogenic Stroke and Underlying AF (CRYSTAL AF) trial, Sanna et al. demonstrated that prolonged rhythm monitoring with implantable loop recorders significantly increased detection of subclinical atrial fibrillation compared with conventional monitoring strategies in patients with embolic syndromes [5].

Identification of an embolic mechanism is particularly relevant when considering secondary prevention strategies. Saver et al. demonstrated improved long-term outcomes with patent foramen ovale closure compared with medical therapy alone in selected patients with cryptogenic embolic events [6]. Additional randomized data from Mas et al. further supported the benefit of patent foramen ovale closure compared with antiplatelet therapy in carefully selected patients [7]. Similarly, Søndergaard et al. confirmed the efficacy of device closure in reducing recurrent embolic events in patients with patent foramen ovale associated with cryptogenic stroke [8].

The concept of coronary embolism as a cause of myocardial infarction was initially described in pathologic studies. Prizel et al. demonstrated that embolic myocardial infarction frequently involved distal coronary vessels and occurred in the absence of significant proximal coronary atherosclerosis [9].

In this report, we present a case of ST elevation myocardial infarction caused by paradoxical coronary embolism through a patent foramen ovale associated with an atrial septal aneurysm. This case highlights the importance of recognizing embolic mechanisms of myocardial infarction and underscores the value of multimodal imaging, rhythm surveillance, and longitudinal follow-up in patients presenting with myocardial infarction and minimal coronary atherosclerosis.

## Case presentation

A 57-year-old man with a history of hyperlipidemia and prior tobacco use presented with sudden-onset substernal chest pain at rest associated with diaphoresis. The pain was described as severe pressure radiating across the chest without clear precipitating factors.

**Figure 1:**
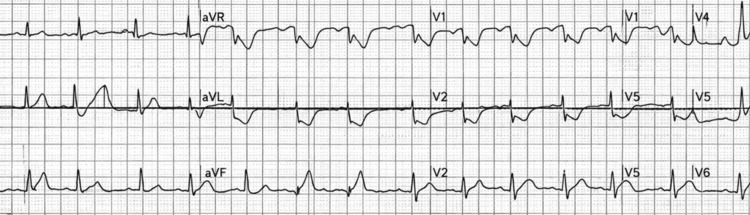
Initial Electrocardiogram Demonstrating Inferior ST Segment Elevation Prehospital ECG demonstrating ST segment elevation in leads II, III, and aVF with lateral extension. ECG: electrocardiogram

An initial electrocardiogram (ECG) obtained by emergency medical services demonstrated ST segment elevation in the inferior leads II, III, and aVF with extension into the lateral leads, consistent with an acute inferior ST elevation myocardial infarction involving the right coronary artery (RCA) territory (Figure 1).

**Figure 2:**
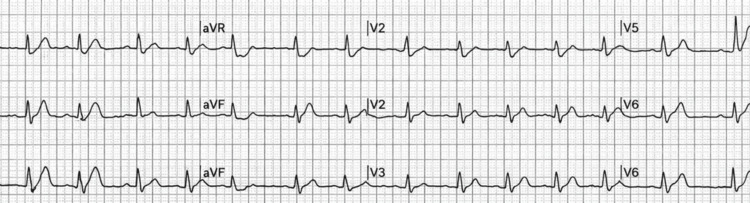
Repeat ECG Demonstrating Partial ST Segment Resolution After Antithrombotic Therapy Repeat ECG after aspirin and intravenous heparin showing partial resolution of the inferior ST segment elevation. ECG: electrocardiogram

Upon arrival at the emergency department, the patient was administered aspirin and started on intravenous heparin according to acute coronary syndrome protocols. A repeat electrocardiogram demonstrated partial improvement in ST segment elevation with persistent inferior ST elevation (Figure 2).

Initial laboratory evaluation demonstrated elevated high-sensitivity troponin, which peaked at 93,311.9 ng/L during hospitalization.

**Figure 3:**
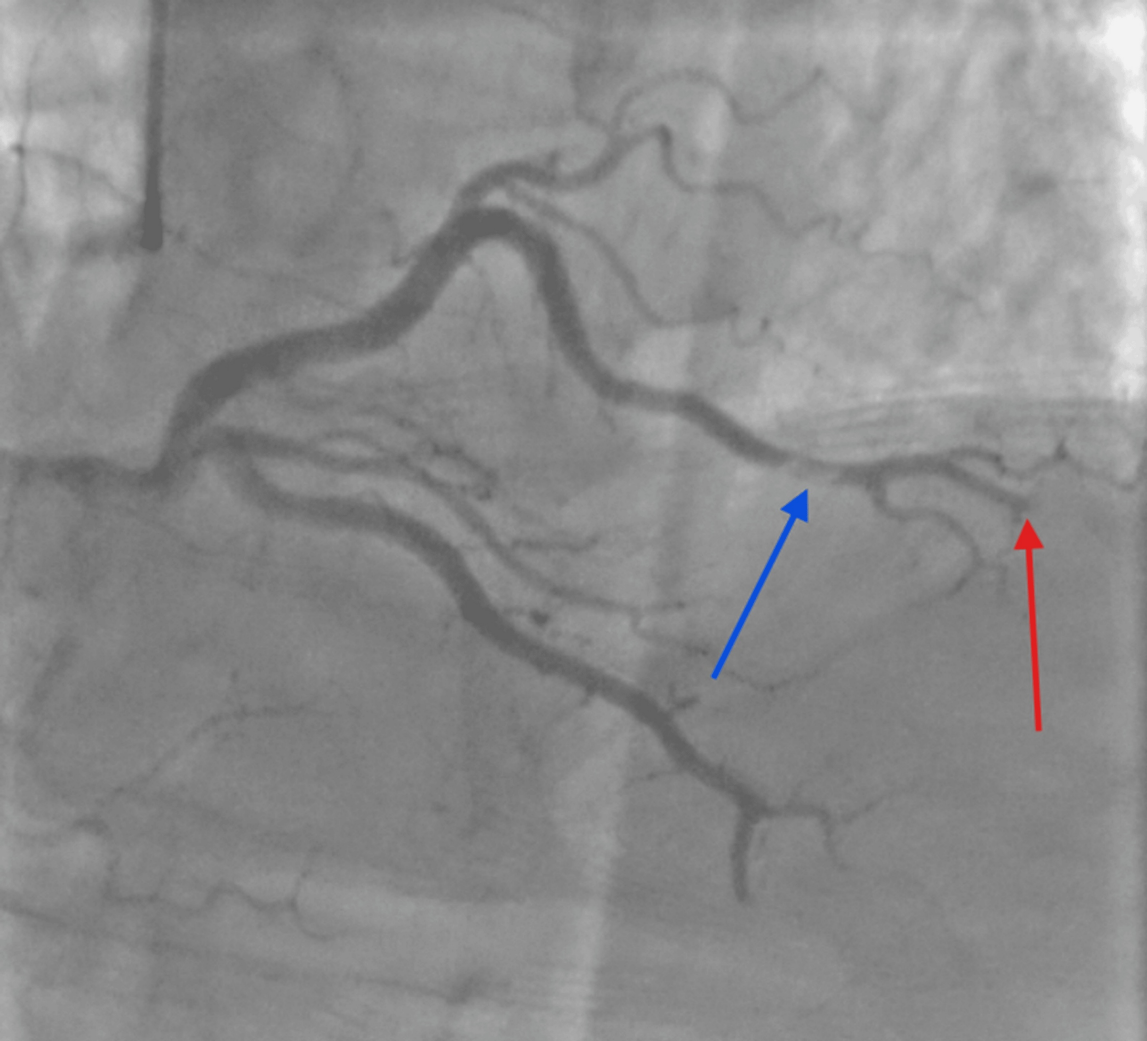
Distal Thrombus in the Posterolateral Branch of the RCA and the Apical Segment of the rPDA Distal thrombus in the posterolateral branch of the RCA and apical rPDA. Right dominant RCA with preserved proximal flow and abrupt distal cutoff. The blue arrow indicates thrombus; the red arrow indicates occlusion. RCA: right coronary artery, rPDA: right posterior descending artery

The patient was taken emergently to the cardiac catheterization laboratory. Coronary angiography demonstrated a dominant right coronary artery supplying the apex via the posterior descending artery, with preserved proximal flow and abrupt distal vessel cutoff associated with a globular filling defect, consistent with distal thrombotic occlusion (Figure 3). Additional projections confirmed occlusion of the distal posterior descending and posterolateral branches, with no significant obstructive disease in the proximal vessels.

**Figure 4:**
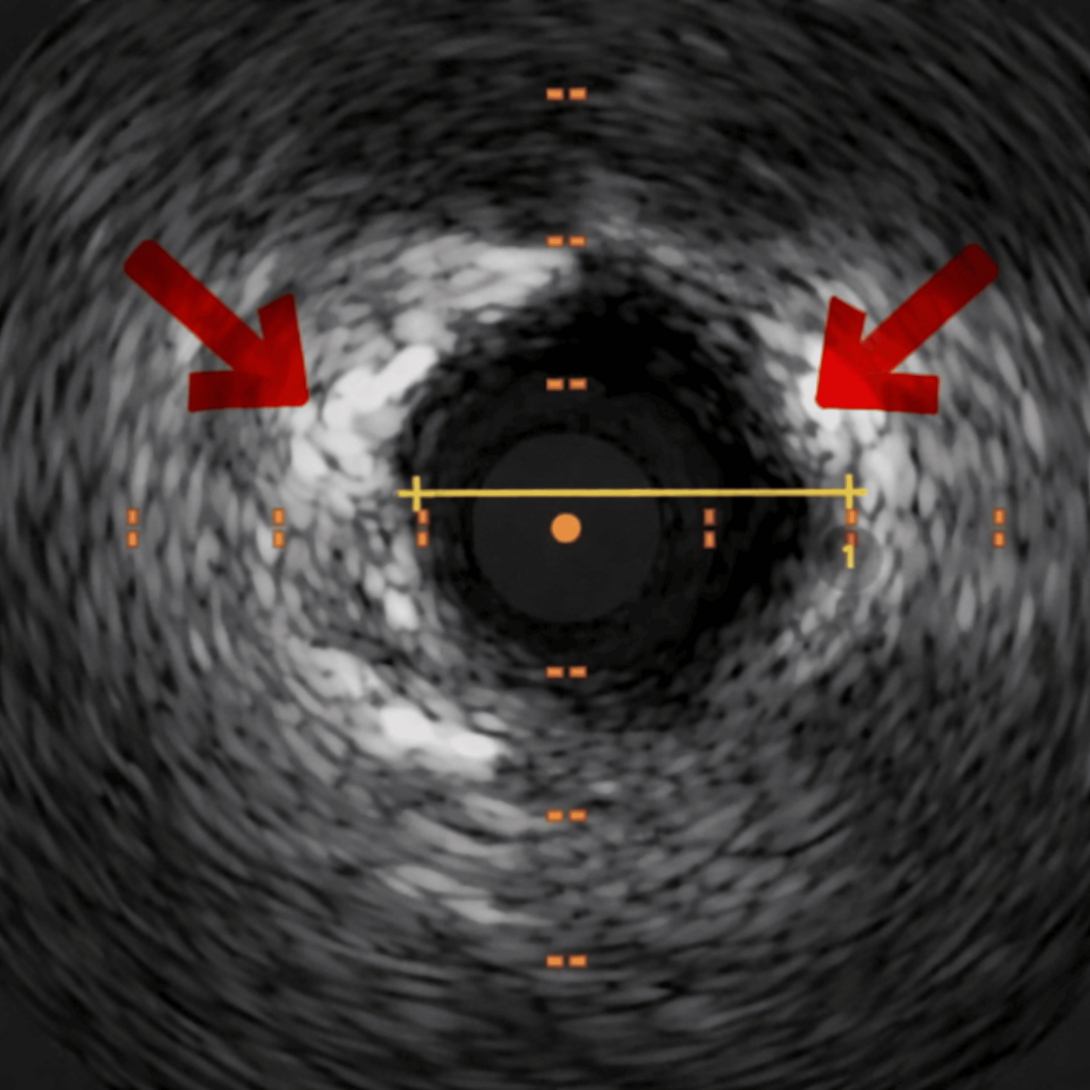
IVUS Demonstrating Absence of Plaque Rupture IVUS demonstrating mild coronary atherosclerosis without evidence of plaque rupture, erosion, or dissection. Red arrows highlight areas of atherosclerotic plaque. IVUS: intravascular ultrasound

Intravascular ultrasound (IVUS) demonstrated mild coronary atherosclerosis without plaque rupture, erosion, or dissection (Figure 4), supporting a non-atherosclerotic mechanism of infarction. While apical wall motion abnormalities raised consideration of distal left anterior descending (LAD) involvement, angiography did not demonstrate obstructive disease or thrombotic occlusion in the LAD, and findings remained most consistent with distal embolic involvement of the RCA territory.

Given the distal location of the thrombus and the absence of unstable plaque, percutaneous coronary intervention was deferred, and the patient was managed medically with integrilin and heparin infusion.

**Figure 5:**
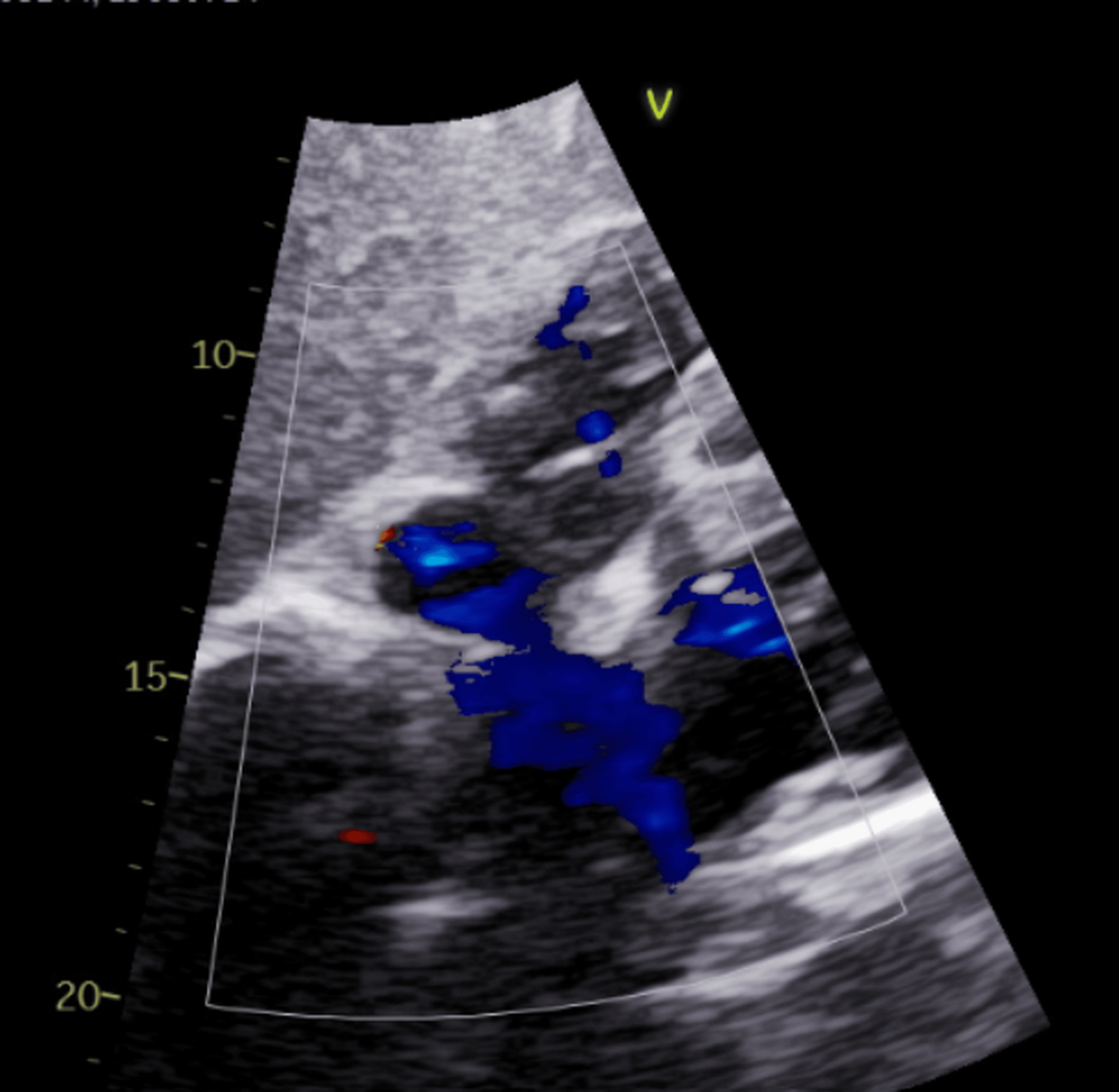
Subcostal Color Doppler Echocardiogram Demonstrating Right-to-Left Shunt Subcostal color Doppler image demonstrating right-to-left interatrial shunting across the atrial septum, with blue color Doppler signal indicating right-to-left flow.

Transthoracic echocardiography demonstrated mildly reduced left ventricular systolic function with an ejection fraction of 45% and regional wall motion abnormalities involving the apex and inferolateral segments, corresponding to the rPDA and posterolateral territories. Color Doppler imaging from the subcostal view suggested right-to-left interatrial shunting (Figure 5), although this modality alone is limited in distinguishing the type of interatrial defect.

**Figure 6:**
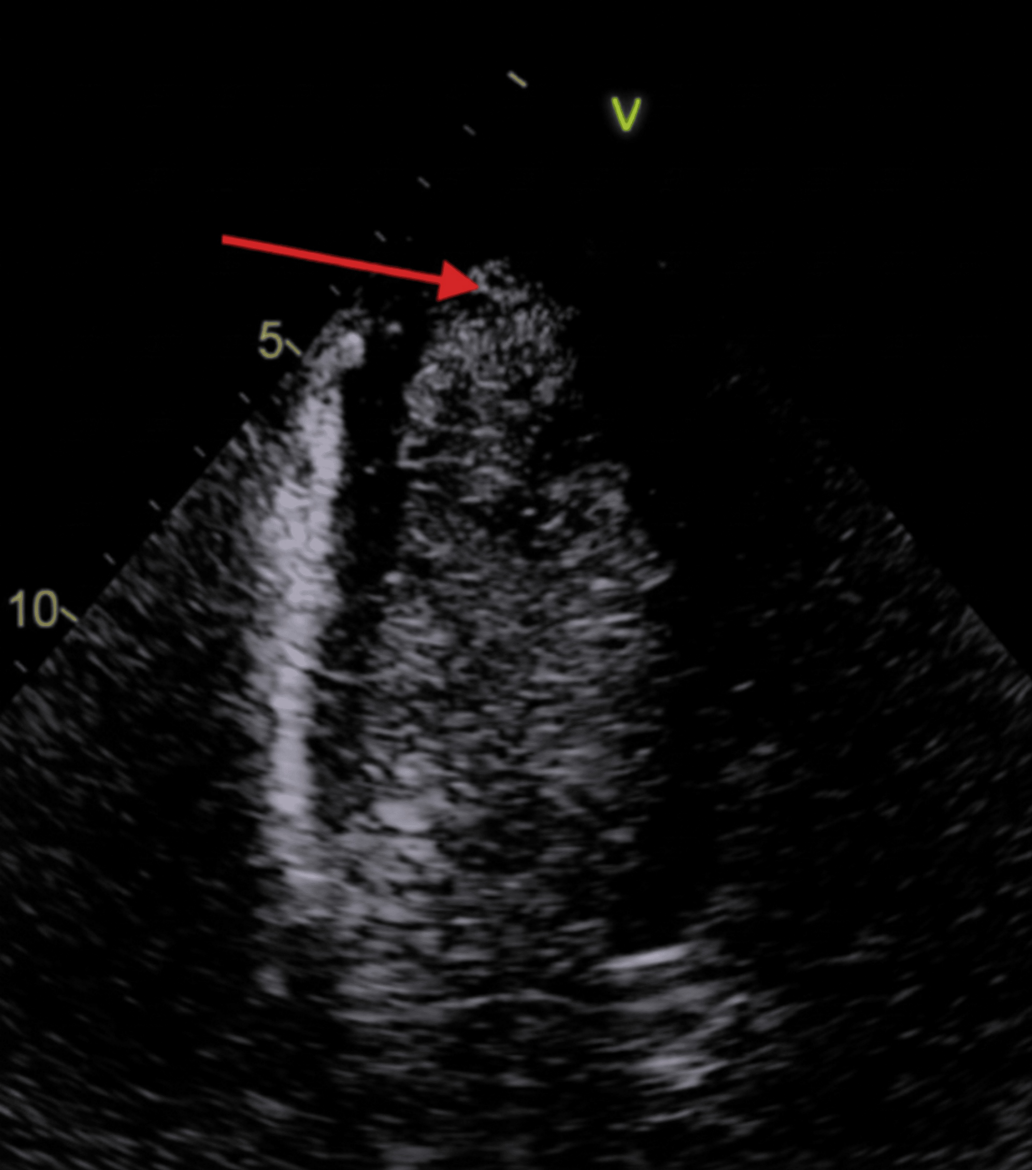
Contrast Transthoracic Echocardiogram Demonstrating Absence of Left Ventricular Thrombus Contrast echocardiogram of the left ventricle with the red arrow indicating the region of interest demonstrating the absence of thrombus.

A contrast transthoracic echocardiogram demonstrated no evidence of left ventricular thrombus at that time (Figure 6).

**Figure 7:**
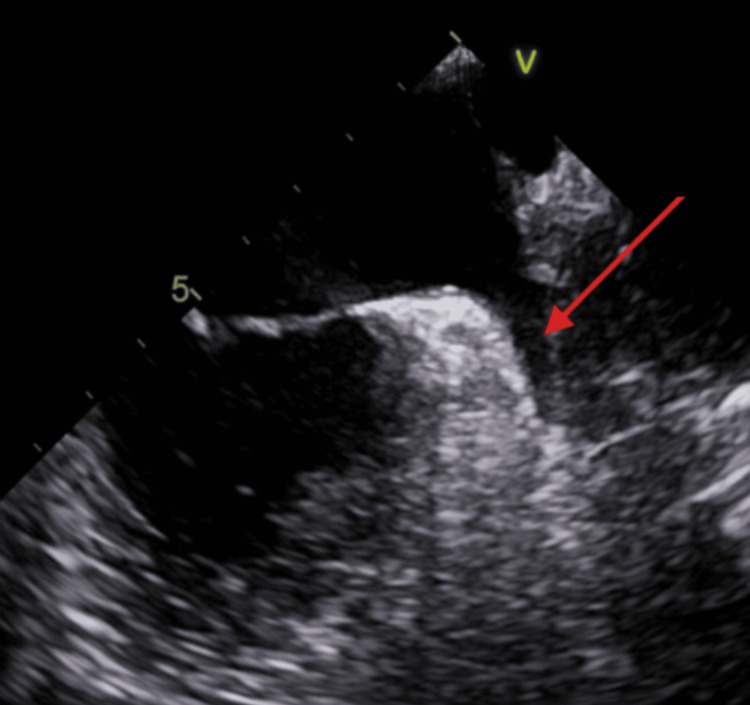
Transesophageal Echocardiographic View of the Left Atrial Appendage TEE image of the left atrial appendage without thrombus, with the red arrow indicating the appendage. TEE: transesophageal echocardiography

**Figure 8:**
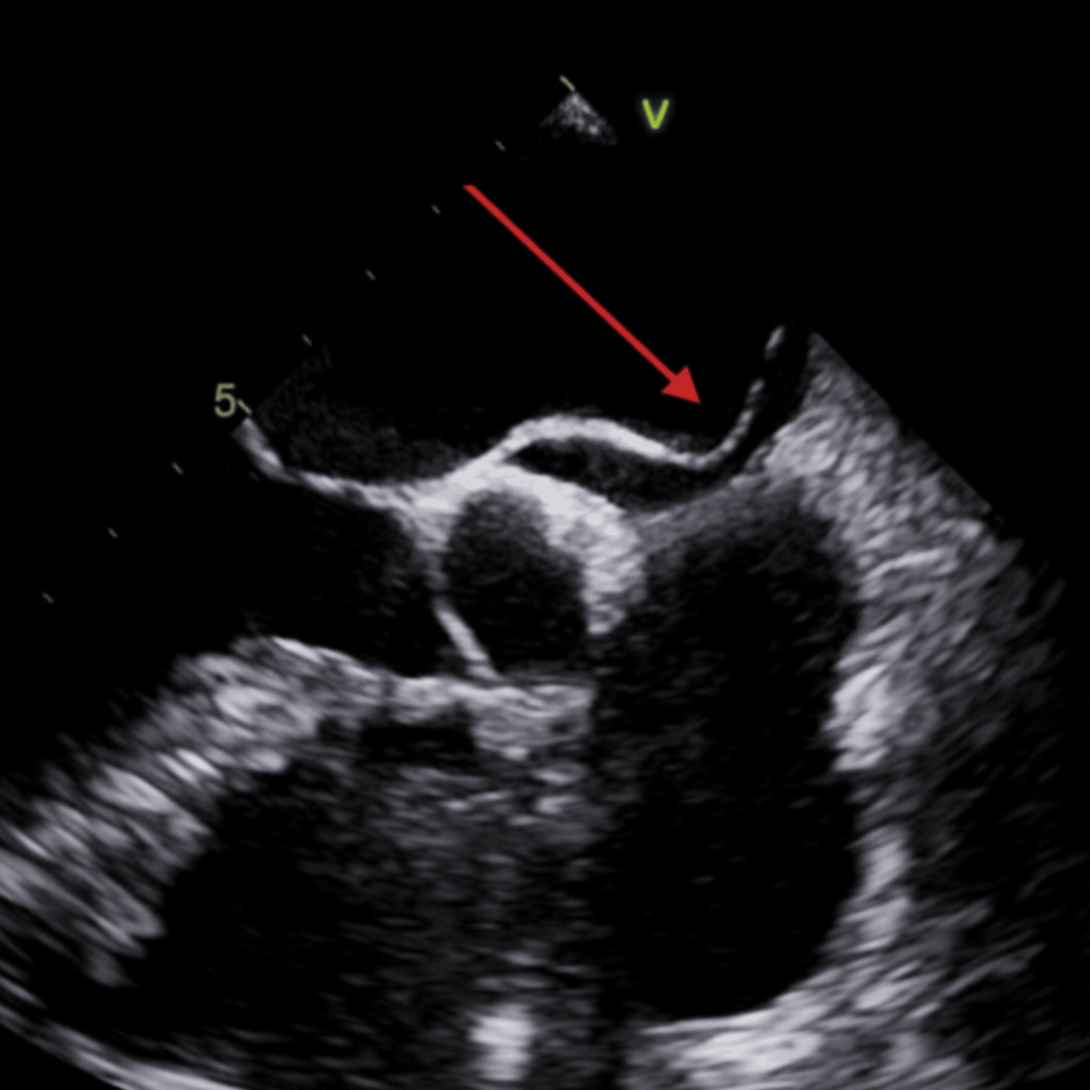
TEE Demonstrating Atrial Septal Aneurysm TEE image demonstrating an aneurysmal interatrial septum with septal deviation toward the right atrium, with the red arrow indicating the aneurysm. TEE: transesophageal echocardiography

**Figure 9:**
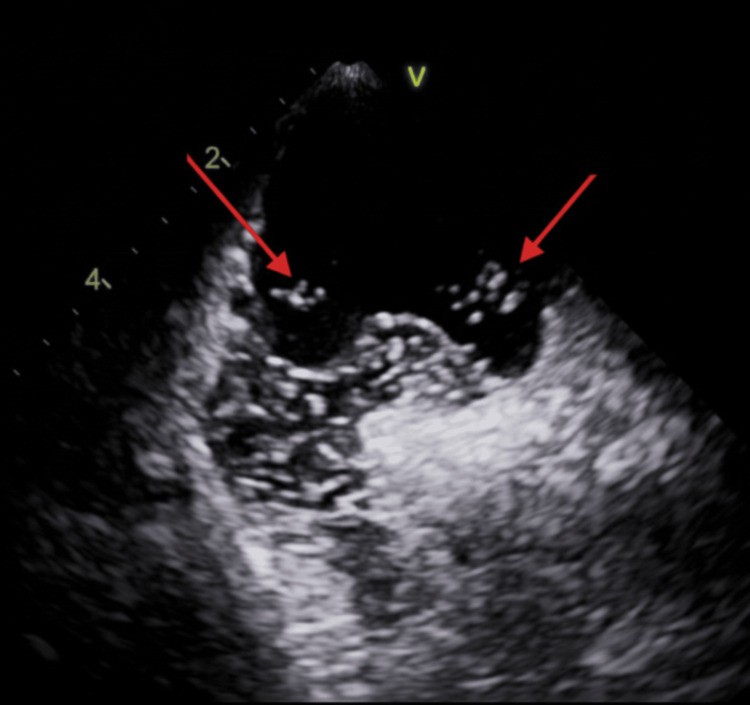
Bubble Study Demonstrating Right-to-Left Intracardiac Shunt Transesophageal echocardiographic bubble study demonstrating microbubbles entering the left atrium shortly after right atrial opacification, confirming right-to-left shunting through a patent foramen ovale, with red arrows indicating the microbubbles.

Further evaluation with transesophageal echocardiography demonstrated no left atrial appendage thrombus (Figure 7), an atrial septal aneurysm (Figure 8), and a bubble study with early right-to-left passage of microbubbles consistent with a patent foramen ovale (Figure 9). No features consistent with a true atrial septal defect were identified, confirming that the diagnosis of interatrial shunt was established based on TEE rather than color Doppler imaging alone.

Lower extremity duplex ultrasound was negative for deep vein thrombosis. Comprehensive hypercoagulable evaluation, including antiphospholipid antibodies, factor V Leiden mutation, and prothrombin gene mutation, was unremarkable. Given concern for occult arrhythmia, an implantable loop recorder was placed on hospital day 2.

The patient was discharged home on hospital day 4 on apixaban, atorvastatin, and metoprolol.

**Figure 10:**
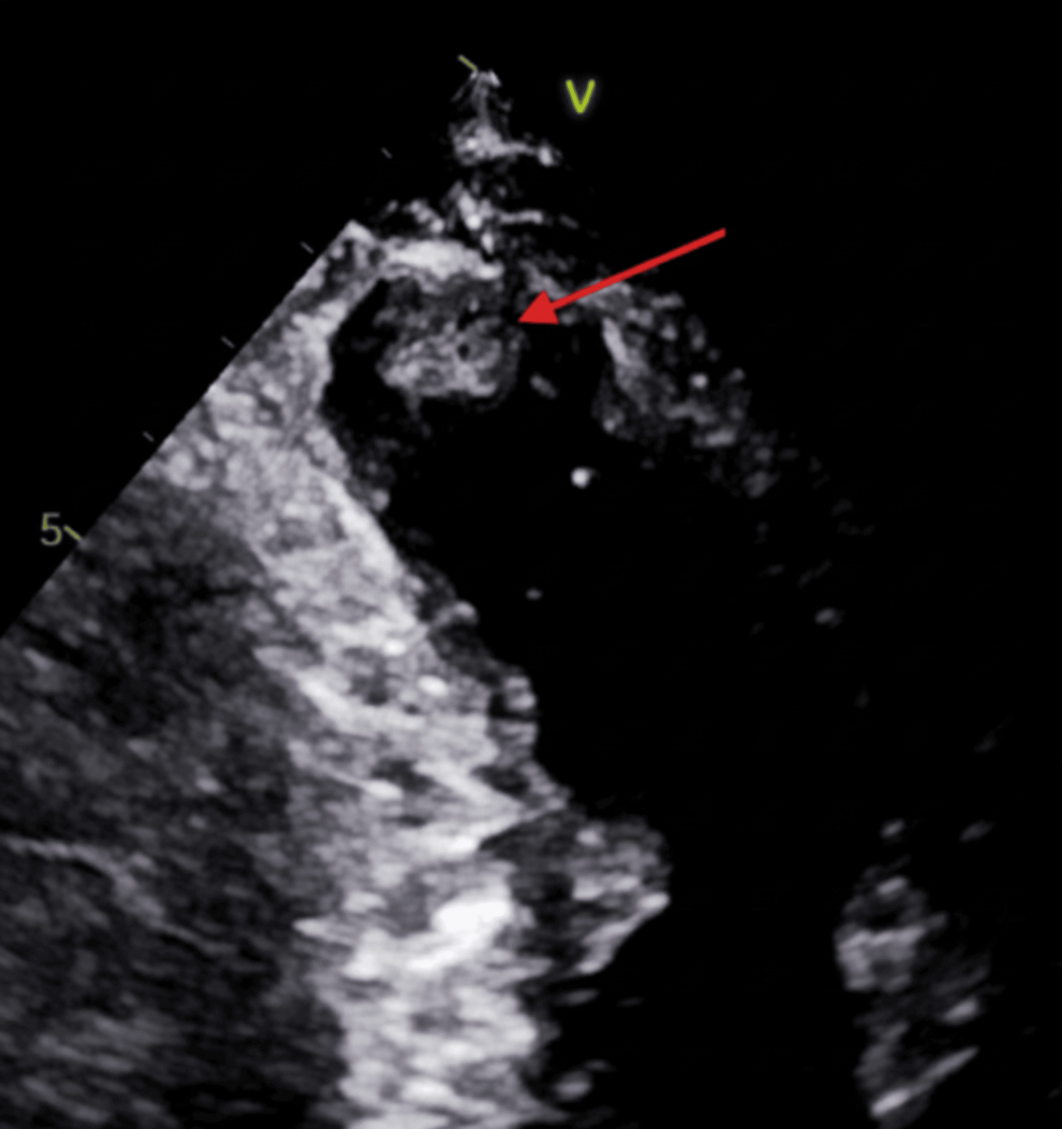
Follow-Up Echocardiogram Demonstrating Apical Aneurysm and Thrombus Formation Follow-up transthoracic echocardiogram demonstrating aneurysmal remodeling of the left ventricular apex, with the red arrow indicating a small apical thrombus.

At follow-up, the patient remained clinically stable without recurrent chest pain, palpitations, or dyspnea. However, repeat transthoracic echocardiography performed on October 15 demonstrated persistent left ventricular dysfunction with aneurysmal remodeling of the apex and development of a small apical thrombus (Figure 10). Notably, the patient reported subtherapeutic anticoagulation due to taking apixaban once daily instead of the prescribed twice-daily dosing. He was counseled extensively on medication adherence and resumed full-dose anticoagulation, with plans for repeat imaging.

The development of left ventricular apical thrombus was therefore most consistent with post-infarction regional wall motion abnormalities and apical akinesis leading to localized blood stasis, despite the presence of anticoagulation.

## Discussion

Embolic myocardial infarction is an underrecognized cause of acute coronary syndrome and may be associated with an increased risk of recurrent embolic events compared with plaque-mediated infarction. Recognition is important, as management and secondary prevention strategies differ from those used in atherosclerotic disease.

Embolic myocardial infarction typically presents with distal coronary occlusion, minimal obstructive coronary artery disease, and preserved proximal flow on angiography [1,2]. The findings in this case are consistent with these features. A comparison of the present case with prior cohort studies is summarized in Table 1.

**Table 1:** Comparison of the Present Case With Prior Studies of Coronary Artery Embolism Comparison of clinical and angiographic features with published cohorts. Findings are consistent with embolic myocardial infarction, with patent foramen ovale identified as the likely source in the absence of atrial fibrillation. MI: myocardial infarction, AMI: acute myocardial infarction, CAD: coronary artery disease, IVUS: intravascular ultrasound, ILR: implantable loop recorder

Feature	Present case	Shibata et al. (2015) [1]	Raphael et al. (2018) [2]
Number of patients	1	177 embolic MI (2.9% of 5,552 AMI)	52 embolic MI
Mean age (years)	57	67 ± 14	62 ± 15
Male sex	Yes	~63%	~60%
Distal coronary occlusion	Yes	≈72%	≈69%
Significant obstructive CAD	No	≈27%	≈35%-40%
Absence of plaque rupture (angiography/IVUS)	Yes (IVUS)	≈73%	Majority
Atrial fibrillation (present or detected)	No (ILR placed)	≈73%	≈58%
Patent foramen ovale identified	Yes	Rarely reported	Rarely reported
Hypercoagulable disorder identified	No	≈6%	Rare
Recurrent embolic events	Pending	≈15% (follow-up)	≈11%-15%
Anticoagulation prescribed	Yes	≈90%	Majority
In-hospital mortality	No	≈8%	≈6%

The present case demonstrates classic features of embolic myocardial infarction while highlighting a less common mechanism. In contrast to prior series in which atrial fibrillation represented the predominant embolic source, no atrial fibrillation was identified despite rhythm monitoring. Instead, a patent foramen ovale with right-to-left shunting was identified, supporting paradoxical embolism as the most likely mechanism.

Paradoxical embolism through a patent foramen ovale is a recognized mechanism of systemic embolization, particularly in the presence of an associated atrial septal aneurysm [3,4]. In this patient, distal coronary embolization, minimal coronary atherosclerosis on intravascular imaging, and intracardiac right-to-left shunting further support this mechanism.

Intravascular imaging is useful in distinguishing embolic occlusion from plaque-mediated infarction when angiographic findings are atypical. In this case, intravascular ultrasound demonstrated no evidence of plaque rupture, erosion, or dissection.

Prolonged rhythm monitoring improves detection of occult atrial fibrillation in patients with embolic events and may influence management [5]. In addition, randomized trials have demonstrated the benefit of patent foramen ovale closure in selected patients with embolic stroke [6-8]. Although data specific to embolic myocardial infarction are limited, identification of an embolic mechanism has important implications for secondary prevention, including anticoagulation, rhythm surveillance, and consideration of structural intervention in selected patients.

## Conclusions

Embolic myocardial infarction should be considered in patients presenting with ST elevation myocardial infarction and minimal or non-obstructive coronary artery disease. Distal coronary occlusion with preserved proximal flow and absence of plaque rupture on intravascular imaging should prompt evaluation for an embolic source.

In this case, multimodal imaging identified findings consistent with paradoxical embolism through a patent foramen ovale with atrial septal aneurysm. Recognition of this mechanism has implications for secondary prevention, including anticoagulation, rhythm monitoring, and consideration of structural intervention in selected patients.
